# Postoperative Malnutrition and Early Pulmonary Metastasis in an Older Patient With Uterine Leiomyosarcoma: A Case Report

**DOI:** 10.7759/cureus.97841

**Published:** 2025-11-26

**Authors:** Harumichi Banno, Daisuke Inoue, Taro Ito, Makoto Orisaka, Yoshio Yoshida

**Affiliations:** 1 Obstetrics and Gynecology, Maizuru-Kyosai Hospital, Maizuru, JPN; 2 Obstetrics and Gynecology, University of Fukui, Fukui, JPN; 3 Obstetrics and Gynecology, University of Fukui, Fukui, JPN

**Keywords:** older cancer patients, postoperative malnutrition, prognostic nutritional index (pni), pulmonary metastasis, uterine leiomyosarcoma

## Abstract

Although the surgical treatment of older cancer patients requires a multifaceted evaluation and appropriate intervention, many problems remain. In this case, we evaluated an 87-year-old patient with stage IB uterine leiomyosarcoma and an appendiceal tumor. The evaluation included a general preoperative examination, geriatric assessment, Clinical Risk Analysis Index for vulnerability screening, and assessment of nutritional status using the prognostic nutritional index (PNI). The patient was deemed operable and underwent a total hysterectomy and appendectomy.

The tumor was completely resected with no unexpected complications. However, the patient developed a paralytic ileus in the early postoperative period. The PNI worsened, and the patient remained in a malnourished state. On postoperative day 17, the patient developed pleural effusion and multiple pulmonary metastases, leading to a transition to best supportive care. This case highlights the critical importance of postoperative nutritional status in older cancer patients.

## Introduction

Uterine sarcoma is a rare and highly aggressive malignant tumor, accounting for approximately 3-7% of all uterine cancers [1]. Although uncommon, its incidence increases with age. An analysis of 13,089 cases from the SEER database demonstrated that 31.6% of patients were aged ≥70 years, and the age-adjusted incidence among women ≥50 years (6.4 per 100,000) was more than four fold higher than that among younger women (1.5 per 100,000) [2]. Because surgical resection remains the only potentially curative treatment [3], perioperative assessment of physiological reserve is essential, particularly in older adults who often present with frailty, multimorbidity, and reduced homeostatic capacity.

Host-related factors, including frailty, immunologic reserve, and nutritional status, play a critical role in determining postoperative outcomes and long-term survival in older cancer patients. Patients can be broadly categorized into three groups: “fit,” “vulnerable,” and “frail.” Fit patients can receive the same standard of care as their younger counterparts, vulnerable patients require appropriately adjusted treatment intensity, and frail patients are best managed with palliative or best supportive care [4,5].

Malnutrition is common in vulnerable or frail older adults, with reported rates ranging from 28% to 70% among patients with gynecologic cancers [6]. Malnutrition impairs cellular immunity, heightens inflammatory responses, increases susceptibility to postoperative complications, and has been associated with reduced treatment tolerance, early recurrence, and decreased survival [7]. Surgery itself induces a transient but substantial immunosuppressive state lasting up to two weeks, during which residual tumor cells may more readily proliferate [8-10]. Postoperative nutritional deterioration can further exacerbate this vulnerability by aggravating lymphopenia and diminishing antitumor immune surveillance [7].

Several methods exist to assess nutritional status, with the prognostic nutritional index (PNI) being a simple yet effective measure that reflects the immunonutritional status of patients [11]. The PNI is useful in various types of cancer [12], and recent studies have identified preoperative PNI levels as an independent prognostic factor in uterine cancer [13]. However, the impact of worsening postoperative PNI values on prognosis in gynecologic cancers remains underreported.

Here, we present the case of an 87-year-old woman with uterine leiomyosarcoma who exhibited marked postoperative nutritional deterioration followed by unusually early pulmonary metastasis. This case highlights an underrecognized but clinically important scenario and underscores the need for vigilant postoperative nutritional monitoring and early intervention in high-risk older adults with uterine sarcoma.

## Case presentation

Baseline characteristics and general health status

An 87-year-old woman, gravida 2 para 2, with a medical history of hypertension and ischemic enterocolitis, had been receiving pessary ring treatment for pelvic organ prolapse for five years. She was functionally independent and lived at home. Her height was 153 cm, weight 55.7 kg, and body mass index (BMI) 23. Her Eastern Cooperative Oncology Group (ECOG) performance status was 0, indicating full activity without restriction.

Comprehensive preoperative assessments were performed to evaluate her frailty and surgical fitness. The Geriatric Assessment (GA) [12] and Clinical Risk Analysis Index (RAI-C) [13] were utilized as recommended for elderly cancer patients. The patient’s G8 score was 13.5, and her RAI-C score was 16, placing her near, but not exceeding, the cutoff thresholds for increased risk. Nutritional evaluation using the PNI [9] yielded a value of 52.51, suggesting preserved immunonutritional status. Taken together, her overall condition was classified as “fit” to “vulnerable,” and she was considered eligible for standard surgical intervention after multidisciplinary evaluation and shared decision-making with her family.

Preoperative findings and diagnosis

**Figure 1:**
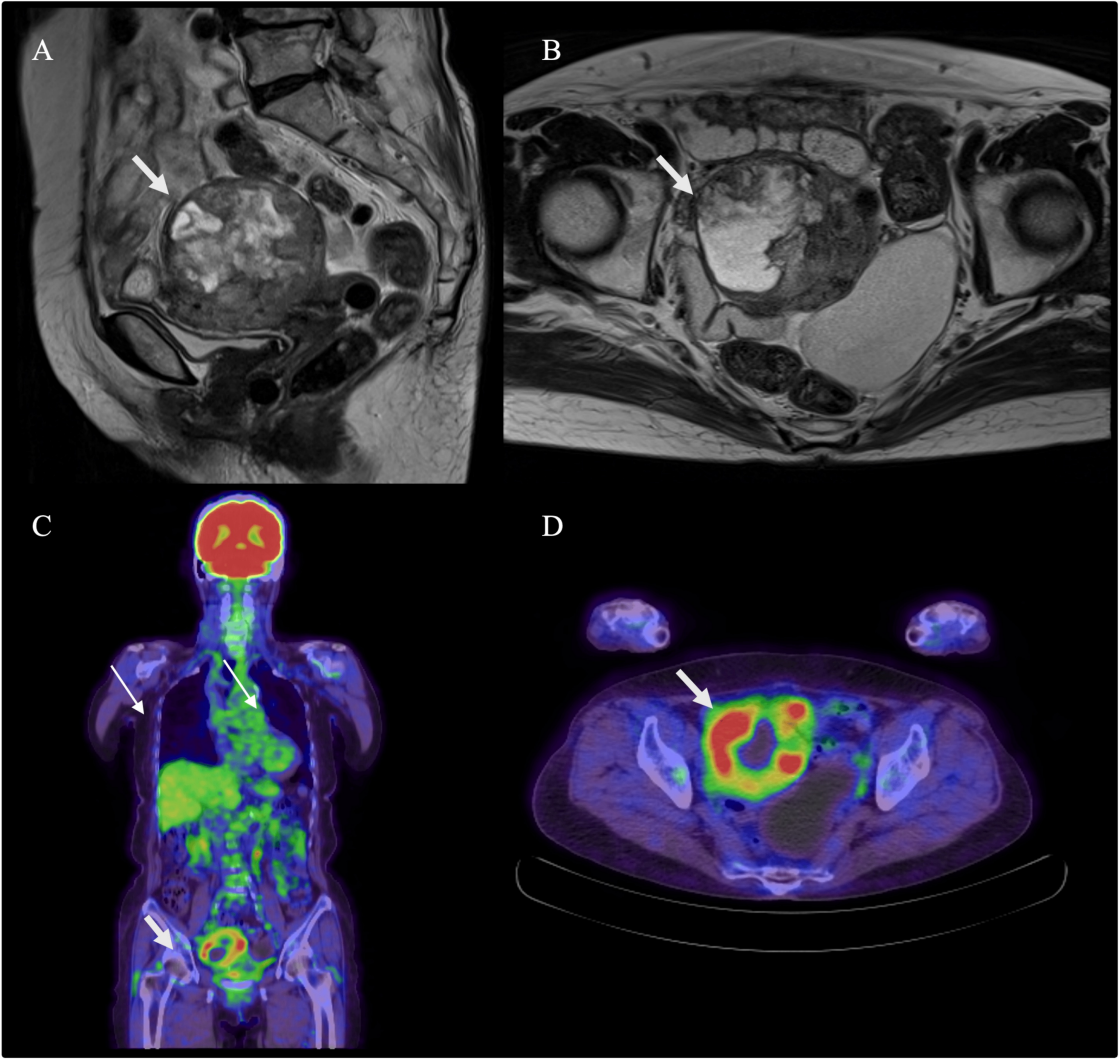
Preoperative MRI and FDG-PET/CT imaging of the uterine tumor A)  Sagittal T2-weighted MRI shows a 7.7 cm intramural uterine mass with heterogeneous signal intensity within the myometrium (thick white arrow). B)  Axial T2-weighted MRI reveals the same lesion with irregular internal signal intensity and indistinct margins. C)  The FDG-PET/CT scan (whole body image) showed strong accumulation of FDG only in the uterine tumor (thick white arrow), but no accumulation in the lung field (thin white arrow). D)  The FDG-PET/CT scan (coronal section) showed strong accumulation of FDG only in the uterine tumor (thick white arrow).

**Figure 2:**
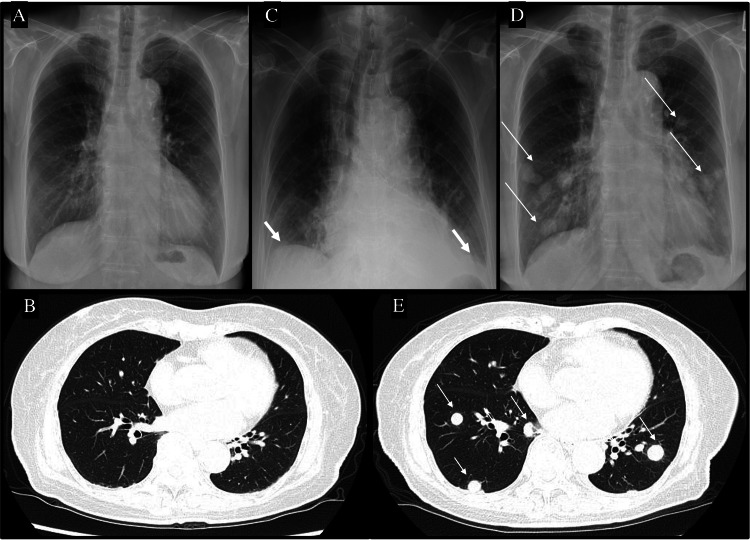
Rapid development of pulmonary metastases following surgery in an elderly uterine sarcoma patient A)    The preoperative chest X-ray showed no abnormal findings. B)    The preoperative chest CT scan showed no abnormal shadows in the lung fields. C)    The chest X-ray on postoperative day 4 showed a Grade 2 pleural effusion (thick white arrow). D)    The chest X-ray on postoperative day 17 showed numerous nodular shadows in the bilateral lung fields, as well as enlarged left hilar and mediastinal lymph nodes (thin white arrows). E)    The emergency chest CT scan on postoperative day 17 revealed numerous nodular shadows in the bilateral lung fields and enlarged left hilar and mediastinal lymph nodes (short thin white arrows).

In November of year X-2, a 3 cm intramuscular uterine mass was incidentally detected during a routine pessary ring follow-up. Surveillance imaging revealed gradual tumor growth, prompting further evaluation. In March X, preoperative work-up included blood tests, chest X-ray, MRI, contrast-enhanced CT, and 18F-fluorodeoxyglucose positron emission tomography (FDG-PET) /CT. Blood tests were within normal limits. MRI identified a 7.7 cm heterogeneous mass in the posterior uterine wall and a 4 cm cystic lesion in the appendix (Figures 1A, 1B). FDG-PET/CT showed intense FDG uptake confined to the uterine mass (Figures 1C, 1D), with no signs of extrauterine spread or pelvic lymph node involvement. Chest X-ray and CT showed no evidence of pulmonary metastasis (Figures 2A, 2B). The clinical diagnosis was uterine sarcoma, classified as T1b (TNM) and Stage IB (FIGO). Surgical resection with curative intent was planned, consisting of total abdominal hysterectomy, bilateral salpingo-oophorectomy, and appendectomy.

Operative course and postoperative progression

In April X, the patient underwent uncomplicated surgery under general and epidural anesthesia. Surgical procedures included a simple total abdominal hysterectomy, bilateral salpingo-oophorectomy, and appendectomy. The operative time was two hours, with an estimated blood loss of 140 mL. Histopathology revealed uterine leiomyosarcoma measuring 7.5 cm, confined to the uterus (pT1b) with no metastases to the adnexa or omentum. The left ovary had a serous cyst and the appendiceal tumor was a low-grade mucinous tumor. The final diagnosis was uterine leiomyosarcoma pT1bNXMX (TNM classification), stage IB (FIGO classification).

Postoperatively, the patient developed paralytic ileus (Clavien-Dindo grade II). Management consisted of fasting and intravenous fluid therapy provided according to the hospital’s standard perioperative care protocol, with input from the institutional nutrition team as part of routine practice. From postoperative days (PODS) 2 to 4, she received isotonic crystalloid solutions and 5% dextrose, without the use of protein-, amino acid-, or lipid-containing formulations. No parenteral nutrition was initiated during this early period, as the ileus was expected to resolve promptly.

**Figure 3:**
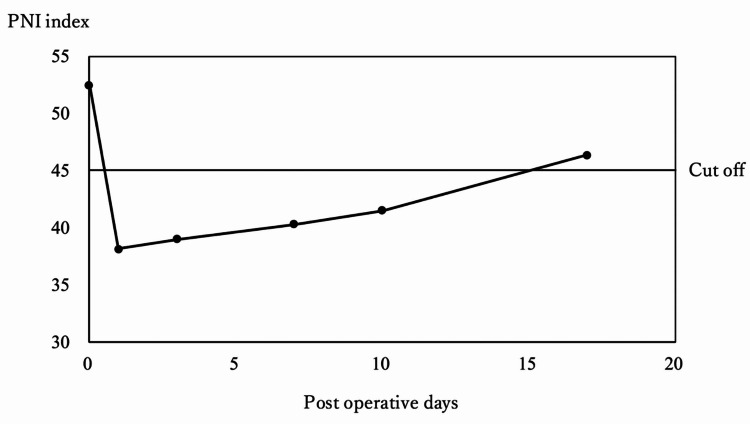
Perioperative changes of PNI values PNI, Prognostic Nutritional Index

Following improvement of bowel function on POD 4, the hospital dietitian assessed the patient and guided the gradual reintroduction of oral intake. The patient progressed from sips of water and clear liquids to a soft diet by POD 8. However, despite this structured dietary advancement, her overall caloric and protein intake remained lower than recommended levels, largely due to reduced appetite and early satiety. No oral nutritional supplements were added during hospitalization. Her postoperative nutritional status, reflected by persistently low PNI values, did not return to preoperative levels by the time of discharge on POD 14 (Figure 3). On postoperative day 16, she became acutely agitated while ambulating and presented to the emergency department the following morning. Although no metastasis was observed on postoperative day 4 (Figure 2C), the subsequent chest X-ray (Figure 2D) and CT scan (Figure 2E) revealed numerous nodular shadows in the bilateral lungs and enlarged left hilar and mediastinal lymph nodes, leading to the diagnosis of pulmonary and lymph node metastatic recurrence of uterine leiomyosarcoma. After a multidisciplinary discussion and consultation with the patient and her family, a palliative care approach was initiated.

This case report was conducted in accordance with the principles of the Declaration of Helsinki. Written informed consent was obtained from the patient.

## Discussion

We report a case of an independent, very old patient with early-stage uterine sarcoma who was considered operable but developed early postoperative paralytic ileus, leading to prolonged deterioration of nutritional status and the early onset of multiple pulmonary metastases.

Surgical resection, while a cornerstone of cancer treatment, can paradoxically create a window of opportunity for metastatic spread of tumor cells, as perioperative immune suppression and tumor-promoting physiological changes have been well documented in several studies [8-10]. This postoperative immunosuppressive state peaks around the third postoperative day and can persist for approximately two weeks [8]. During this critical period, patients may be particularly vulnerable, and, as suggested in previous studies, postoperative complications and deterioration of nutritional status can further compromise immune recovery and have been associated with poorer prognosis [14,15].

Postoperative nutritional status could impact the patient's immune system and contribute to progressive cancer activity. Previously, the importance of preoperative nutritional assessment has been emphasized in terms of prognostication. There are two major types of assessments: Subjective Global Assessment (SGA) and Full Nutritional Assessment (FNA). However, due to the complexity and large number of assessment items, they are challenging to apply in real clinical practice [16,17].

The PNI, calculated from serum albumin and total peripheral lymphocyte count, serves as a simplified but effective method for evaluating nutritional status [11]. Lymphocytes, the main effectors of the immune system, play a critical role in tumor elimination, preventing tumor development and metastasis. A decrease in lymphocytes indicates reduced anti-tumor functions [18]. Serum albumin levels reflect nutritional status, as malnutrition is associated with impaired immune function and poor prognosis in malignancies [19]. Both lymphocytes and albumin are immune-related, making PNI a valuable tool for evaluation. Although there are few studies on postoperative nutritional evaluation and prognosis in older cancer patients, the PNI has been associated with survival outcomes in gastrointestinal cancers [20].

Considering these findings, the patient's early postoperative paralytic ileus likely exacerbated the immunosuppressive state, contributing to the rapid onset of pulmonary metastases. This underscores the need for vigilant postoperative nutritional and immune function monitoring.

However, it is important to acknowledge that while PNI is a valuable tool, it is not without limitations. The reliance on serum albumin and lymphocyte counts may not capture all aspects of a patient's nutritional and immune status. Therefore, further research is needed to validate the use of the PNI across different cancer types and patient populations. Additionally, studies exploring the integration of the PNI into routine postoperative care and its impact on long-term survival outcomes are warranted. Such research could help establish standardized guidelines for nutritional interventions in the perioperative management of cancer patients.

## Conclusions

In conclusion, our case study demonstrates that the PNI can be an objective indicator for postoperative management, especially in older cancer patients. Since the PNI is based on serum albumin levels and peripheral lymphocyte counts routinely measured before surgery, it provides a straightforward method for evaluating nutritional status.

Cancer patients with a low PNI should be carefully assessed preoperatively, with particular attention to older patients with aggressive malignant tumors, who may be at higher risk for poor outcomes if their postoperative PNI remains low. Early and targeted nutritional support for these patients could potentially improve their prognosis.
